# Around the Hospitals

**Published:** 1893-12-09

**Authors:** 


### Around the Hospitals.

Notice to the Managers of Institutions.—Special space is now reserved for the insertion of notices of hospital meetings and festivals. The Editor requests that all notices may reach The Hospital Office, 428, Strand, at least one week before the date of each meeting.

**The Nottingham General Infirmary.**—Important changes in the constitution of the governing body of this hospital have been made since last year. The board as now constituted is to consist of a general board electing a monthly board, to consist of governors and members of the medical staff. The monthly board on its part electing a weekly house committee, and a quarterly medical board. This last to report of medical matters to the monthlyboard with liberty to address the general board also when they desire to do so, The hospital shows an increasing activity in other directions. Patients are increasing, whilst the expenditure shows diminution. The result of a canvass by the ladies of Nottingham has added over £800 to the funds, and a like canvass of the country districts is contemplated. It is also proposed to abolish the recommendations in regard to out-patients. The excellent services of Miss Knight and her staff of nurses is acknowledged. The convalescent home provided through Colonel Seeley's generosity adds greatly to the good work done by the hospital. At the anniversary service in St. Mary's Church about £140 was collected. These anniversary services and the importance which they lend to the hospitals, is of great value; it is to be hoped that where they are customary they will continue to prevail, and that more hospitals in our larger towns will benefit by adopting a like proceedure.

**The Salford Royal Hospital.**—The Salford Hospital, in spite of strenuous efforts on its behalf, has not yet tided over its bad days. The income during the past year has failed by £736 to cover the expenditure, and a complete canvass of the district has on the whole yielded unsatisfactory results. In spite of this dispiriting condition of affairs, the institution is steadily progressing. The new departure abolishing letters of recommendation has brought increasing numbers to the hospital. Yet the expenditure is actually less than previously. The utmost care is now taken that help should reach those who need it most, this of course entails extra work. The number of patients benefited during the past year was 20,000, 1,229 of whom were in-patients. Several good legacies have fallen to the lot of the institution recently, including £3,000 from the late Mr. Oliver Heywood, a good friend and former president of the institution, and £1,000 through the will of the late Mr. James Chadwick. To commemorate Mr. Heywood's memory a memorial tablet has been placed in the entrance hall and a ward called after him, and a bed has been called after Mr. Chadwick.

